# Metagenomics enables the first detection of *Trypanosoma* sp. in Streblidae (Diptera: *Hippoboscoidea*) parasitizing bats in São Paulo, Brazil

**DOI:** 10.3389/fsysb.2025.1721019

**Published:** 2026-01-09

**Authors:** Roberta Marcatti, Lucas Augusto Moysés Franco, Esmenia Coelho Rocha, Marcello Schiavo Nardi, Juliana Laurito Summa, Eric Thal Brambilla Cordeiro da Silva, Adriana Ruckert da Rosa, Débora Cardoso de Oliveira, Gustavo Graciolli, Ester Cerdeira Sabino

**Affiliations:** 1 Institute of Tropical Medicine (IMT), Faculty of Medicine, University of São Paulo (FMUSP), São Paulo, Brazil; 2 São Paulo Municipal Secretariat for the Environment and Green Areas, São Paulo, Brazil; 3 São Paulo Municipal Health Department, São Paulo, Brazil; 4 Institute of Biosciences, Federal University of Mato Grosso do Sul (UFMS), Campo Grande, MS, Brazil

**Keywords:** bat flies, bats, metagenomics, neobat trypanosoma, phylogeny, streblidae, bioinformatics

## Abstract

**Introduction:**

Bats play important ecological roles but can also harbor a wide diversity of pathogens, including trypanosomatids. Knowledge about the circulation of Trypanosoma spp. in bat ectoparasites remains limited, particularly in peri-urban environments.

**Methods:**

In this study, we used shotgun metagenomic sequencing to investigate the presence of Trypanosoma spp. in streblid flies parasitizing Carollia perspicillata bats collected in a peri-urban fragment of the Atlantic Forest in São Paulo, Brazil. A small, preliminary set of pooled samples was analyzed, followed by phylogenetic reconstruction.

**Results:**

Trypanosoma sequences were detected in flies from the family Streblidae. Phylogenetic analysis showed that these sequences cluster within the Neobat 4 clade, which has previously been reported in Carollia spp. bats. This represents the first detection of Trypanosoma sp. in streblid flies parasitizing bats in São Paulo.

**Discussion:**

Although the vector competence of streblid flies for Trypanosoma transmission is still unknown, their close ecological association with bats suggests that they may serve as a non-invasive tool for pathogen surveillance when direct bat sampling is limited. This study expands the known geographic distribution of the Neobat 4 clade and contributes to understanding parasite circulation among bats and their ectoparasites.

## Introduction

Bats are flying mammals distributed on all continents except Antarctica and represent around 20% of all mammal species, with over 1,400 known species in total and their origin is estimated to have occurred during the Eocene, around 60 million years ago (Lei and Dong, 2016). They provide numerous ecosystem services and their preservation is extremely important for the maintenance of green areas (Kunz et al., 2011). Despite their undeniable ecological importance, bats are also considered reservoirs of numerous infectious agents of importance to Public Health, including viruses (rabies, coronaviruses, paramyxoviruses), bacteria (*Bartonella*, *Leptospira*), and parasites (trypanosomatids, *Plasmodium*, *Leishmania*), highlighting the need for continuous pathogen surveillance to better understand the spatial distribution of these agents in natural areas, especially within the current scenario of intense climate change (Han et al., 2016).

These mammals maintain intrinsic relationships with hematophagous ectoparasites specific to this animal group: dipterans of the Families Streblide and Nycteribiidae. Although both have evolved in close association with bats, specific co-evolution with host species has resulted in distinct morphological adaptations for each group. This close relationship and the resulting adaptations make these dipterans excellent models for the study of host-parasite co-evolution (Dick and Patterson, 2006). These flies exhibit remarkable host specificity and employ reproductive strategies that maximize fitness, including host switching within colonies (Sebastián Tello et al., 2008; Graciolli and Dick, 2004; Morse et al., 2012). Such behavioral characteristics, combined with their obligate hematophagy and intimate contact with bat hosts, position these ectoparasites as potential players in pathogen transmission dynamics. The high host fidelity observed in many streblid species, coupled with their ability to move between individual hosts within roosting sites, creates opportunities for both vertical transmission (parent to offspring through the pupiparium) and horizontal transmission (between hosts) of pathogens. Additionally, the longevity of adult flies and their repeated blood meals throughout their lifecycle may facilitate pathogen acquisition, amplification, and dissemination within bat colonies. Dick and Dittmar, (2014) suggest these ecological and biological characteristics are key factors in understanding pathogen transmission networks in bat-ectoparasite systems (Dick and Dittmar, 2014). The Streblidae family includes about 230 species, mainly found in tropical regions (Patterson et al., 2007), of which 101 species distributed in 24 genera have been recorded in Brazil (Graciolli, 2025).

Bats are natural reservoirs of trypanosomatids from the cruzi clade and possibly contributed to their dispersal between continents (Lima et al., 2015). The *Trypanosomatidae* family includes the genera *Trypanosoma*, *Leishmania*, and *Crithidia*, which are known to cause diseases in various hosts (Lukeš et al., 2018). The genus *Trypanosoma* causes two diseases of great medical relevance: Chagas disease (*Trypanosoma cruzi*) and African sleeping sickness (*Trypanosoma brucei*). Transmission occurs mainly through hematophagous invertebrate vectors, with triatomines, popularly known as kissing bugs, being the main vector (Patterson et al., 2007). In Brazil, infections by *T. cruzi*, *T. rangeli*, *T. c. marinkellei*, *T. dionisii*, and *T. wauwau* have been identified in bats (França et al., 2024; Dario et al., 2017a).

While trypanosomatid detection in streblid flies has not been previously reported, other blood parasites have been documented in bat ectoparasites. Studies have detected *Bartonella* DNA in bat flies, suggesting their potential role as vectors or mechanical transmitters of bacterial pathogens within bat populations (Morse et al., 2012). Additionally, molecular evidence of *Hepatozoon* and other apicomplexan parasites has been found in nycteribiid flies, reinforcing the hypothesis that bat ectoparasites may serve as reservoirs or vectors for diverse hemoparasites (Szentiványi et al., 2020). These findings collectively emphasize the importance of investigating bat flies as potential sentinels for blood-borne pathogens circulating in bat populations.

The strong host-dependence of these bat flies, which rely on bats for feeding and reproduction, makes them useful as potential sentinels for infectious agents. For this reason, an exploratory metagenomic study in such flies was conducted, since this Next Generation Sequencing molecular technique allows the identification of several microorganisms contained in a sample, without the need for prior knowledge of the agents of interest (Wylezich et al., 2020). The objective of this study is to report the first detection and preliminary genetic characterization of *Trypanosoma* sp. in Streblidae flies parasitizing bats, contributing to knowledge about the parasite’s distribution in peri-urban ecosystems in São Paulo municipality.

## Materials and methods

This was an exploratory metagenomic study based on samples of bat ectoparasites (Streblidae) collected in São Paulo, Brazil, in 2022, aiming to characterize potential parasite DNA sequences.

### Study area

**FIGURE 1:**
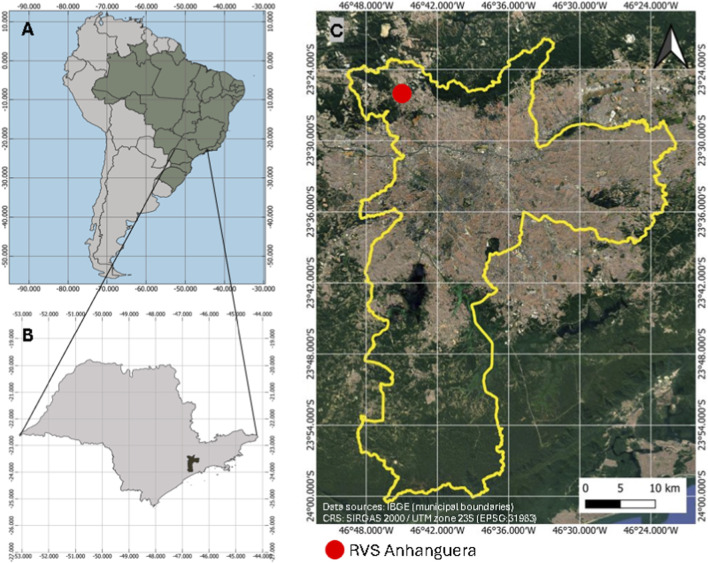
Spatial context of the study area. **(A)** Map of Brazil with the state of São Paulo. **(B)** Location of the Municipality of São Paulo within the state. **(C)** Detailed map of the Municipality of São Paulo and RVS Anhanguera indicated in red.

Samples were collected in August 2022 at the Anhanguera Wildlife Refuge (RVS Anhanguera), located in the Perus district, North Zone of São Paulo (Figure 1), approximately 38 km from the city center (lat. −23° 23’54.69”S, long. −46° 47’27.24”W). With about 800 ha, the area is part of the Northern Atlantic Forest Ecological Corridor and is a priority in the Municipal Plan for Atlantic Forest Conservation and Recovery - PMMA São Paulo. The habitat consists of secondary forest in regeneration, previously used as a eucalyptus plantation, and is continuous with preserved fragments of native Atlantic Forest. The map was generated in QGIS (v3.16) using shapefiles from IBGE (2025).

### Capture and sample preparation

Bats were captured at ground level, beneath the forest canopy, using 10-m-long mist nets with 50mm mesh, set up at dusk and checked every 30 min from 18:00 to 23:00 each night. After capture, bats were temporarily held in cloth bags until processing. Following identification and sample collection, all bats were safely released back into the wild at the site of capture. Streblidae flies parasitizing bats were collected and individually assigned the same identification number as their respective host to maintain host tracking. Flies were stored in dry cryotubes at −80 °C and species identification was performed later in the laboratory, on a cold table at −80 °C, without the use of any preservative buffer. The specimens (bats and flies) were identified to species level. Bat and fly species were identified based on morphological characteristics using taxonomic keys described by Gregorin and Taddei (2002) plus Gardner (2007) and Graciolli and Barros De Carvalho (2025), respectively. No molecular identification was performed. When multiple flies of the same species were collected from a single host, they were processed in pools. After pre-treatment to remove external contaminants with 70% ethanol for 10 s and three rinses in DNA/RNA-free water, the specimens were macerated with 350 µL of phosphate-buffered saline (PBS) and 3 mm stainless steel beads in the L-beader 6 cell disruptor (Loccus, São Paulo, Brazil). After centrifugation at 10,000 RPM for 10 min at 19 °C, the supernatant was processed through a 0.2-micron filter (Merck, Darmstadt, Germany). For every 150 µL of sample, 7.5 µL of Proteinase K (Loccus, São Paulo, Brazil) was added, followed by incubation at 37° for 10 min in a dry bath. DNA and RNA extraction was performed using Extracta - DNA and RNA Viral (Loccus, São Paulo, Brazil), following the manufacturer’s protocol.

### Metagenomics protocol

The SMART-9N protocol (Claro et al., 2023) was used for metagenomic sequencing. In brief, genetic material extracted from samples was treated with Turbo-DNase (Thermo Fisher Scientific, Massachusetts, USA), purified using the Zymo RNA Clean-up & Concentrator-5 kit (Zymo Research, California, USA), and cDNA was synthesized using SuperScript IV (Thermo Fisher Scientific, Massachusetts, USA). Amplification was performed with Q5 Hot Start High-Fidelity 2X Master Mix (New England Biolabs Massachusetts, USA) with random nonamer primers (9N), as described in the SMART-9N protocol, which enables unbiased amplification of cDNA without targeting specific taxonomic groups. DNA concentration was measured using the Qubit 4 Fluorometer (Thermo Fisher Scientific), and samples with a minimum of 50 ng of input DNA were used for sequencing. Sequencing libraries were prepared using the Native barcoding V14 kit (Oxford Nanopore Technologies, Oxford, UK) and sequenced on GridION (Oxford Nanopore Technologies, Oxford, UK) using the R10.4.1 flow cell (Oxford Nanopore Technologies, Oxford, UK). FAST5 files were converted to FASTQ for bioinformatic analysis.

### Bioinformatics

FASTQ sequences were processed in Geneious Prime software (version 2024.1.1). Quality trimming was performed using BBDuk (version 39.06), followed by removal of reads mapping to the human genome and to the ectoparasite host genome. After filtering, reads longer than 500 bp were subjected to an initial taxonomic screening using a mapping-based approach implemented in Geneious and compared against the NCBI nt database. After this metagenomic screening step, all reads preliminarily classified as *Trypanosoma* were extracted and subjected to BLASTn searches (query coverage >70%) to validate their taxonomic identity, with validation by BLASTn at NCBI (query cover >70%) and the *Trypanosoma* species showing the highest similarity to the classified reads was selected as the reference for the final alignment step. Two negative controls were included throughout extraction, library preparation, and sequencing, and any taxa detected in these controls were considered contaminants and removed from all downstream analyses.

Contig assembly was performed in Tadpole (BBTools; version 39.06), and the resulting contigs were aligned to the 18S rRNA gene region using Minimap2 (version 2.26). This region, located within the small ribosomal subunit, is frequently used for phylogenetic studies and species identification. Descriptive statistics and relative frequencies were calculated to support species-level classification of the obtained sequences.

Phylogenetic inference was performed using MEGA12 software in two steps. First, a Maximum Likelihood tree (Tree 1) was inferred with 1000 bootstrap replications. Model selection identified TN93 + G (Tamura–Nei model with Gamma-distributed rate variation) as the best-fit evolutionary model for this dataset, which showed the lowest BIC value (BIC = 18,651.834) among the 24 nucleotide substitution models evaluated. This tree (Figure 3) included the three sequences obtained in this study plus 17 sequences representing described *Trypanosoma* species from the *T. cruzi* clade, all retrieved from GenBank. Accession numbers for all reference sequences are provided in Supplementary Table S1.

In the second step, an additional Maximum Likelihood tree (Tree 2) was generated to examine the placement of the newly obtained *Trypanosoma* sequences relative to previously reported unclassified bat-associated isolates. This tree was generated using the Maximum Likelihood method, with 1000 bootstrap replicates. In MEGA 12, the best-fit substitution model was Tamura–Nei + I, selected based on the lowest BIC value (BIC = 4908.749) among the 24 nucleotide substitution models evaluated by the software. This strategy was used to explore the diversity and evolutionary relationships of bat-associated *Trypanosoma* species in Brazil (Figure 4). The dataset included the three consensus sequences generated in this study and other 26 identified *Trypanosoma* species and multiple unclassified *Trypanosoma* sp. Isolates previously detected in bats; all retrieved from GenBank. Accession numbers for these sequences are also listed in Supplementary Table S1.

Trees were rooted using *Trypanosoma brucei rhodesiense* and a reptile-associated *Trypanosoma* lineage (*Trypanosoma* sp. from gecko). These outgroups were selected because they represent early diverging clades within the genus. Their phylogenetically distant position allows stable root placement and avoids artificial clustering among mammal-associated taxa, thereby improving the reliability of the inferred topology.

The samples analyzed were collected on a single sampling day and were selected based on prior metagenomic findings that indicated the presence of trypanosomes. Samples from other days, in which no trypanosome sequences were detected, were not included in this analysis. As this is a preliminary dataset with a small sample size, no statistical association analyses were performed with other variables of interest.

### Ethics approval and consent to participate

The project was approved by the Ethics Committee of the USP Faculty of Medicine (protocol No. 1439/2020), with a license for animal capture issued by the Secretariat of the Environment and Green Areas, São Paulo City Hall, SP, Brazil, responsible for wildlife management in the municipality.

## Results

**FIGURE 2:**
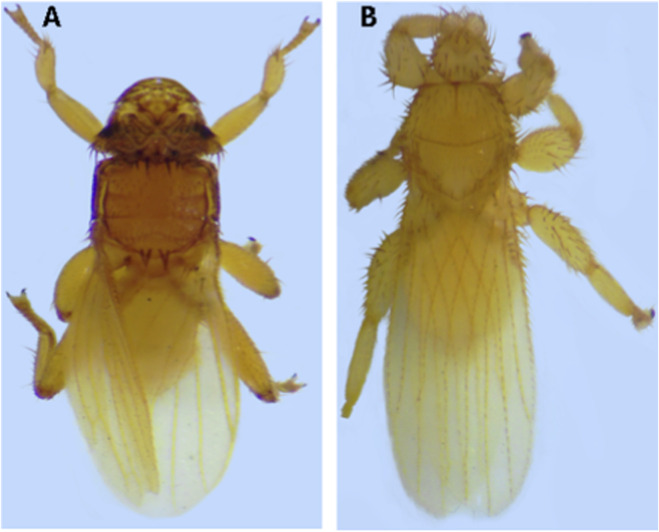
Photographs of bat flies: **(A)** *Strebla guajiro* and **(B)** *Trichobius joblingi*.

Among the 19 bats captured on the sampling day, six were parasitized with Streblidae (31.6%). Analysis of the six specimens revealed that three of them contained sequences of the genus *Trypanosoma* (Table 1). Representative images of the bat flies collected are shown in Figure 2.

**TABLE 1:** Bat species captured, their respective ectoparasitic flies (Streblidae) and number of *Trypanosoma* reads detected by metagenomics.

Bat species	Sex	Age	Streblidae species (n especimens)	n Trypanosoma sp. reads
*Anoura caudifer*	M	A	*Trichobius tiptoni (1)*	—
*Carollia perspicillata*	F	A	*Paraeuctenodes similis (1)*	—
			*Trichobius joblingi (2)*	
*Carollia perspicillata*	F	A	*Strebla guajiro (1)*	388
*Carollia perspicillata*	F	A	*Trichobius joblingi (1)*	—
*Carollia perspicillata*	F	A	*Strebla guajiro (1)*	2448
*Carollia perspicillata*	F	A	*Strebla guajiro (1)*	317

The reads used for taxonomic classification are described in Table 2. Only reads longer than 500 bp were considered to ensure sufficient sequence length for reliable BLAST classification and to minimize false-positive identifications. Among the three samples that tested positive for *Trypanosoma*, samples 3 and 6 each showed 20 reads exceeding 500 bp, whereas sample 5 displayed roughly a seven-fold increase in reads above 500 bp. For read homogeneity, 60 reads from sample “5” were randomly selected, while 20 reads from each of the other samples were selected for BLASTn (Megablast) analysis. Only reads with query cover above 70% were considered for species-level classification.

**TABLE 2:** Absolute number of *Trypanosoma* reads detected by metagenomics.

Sample	Reads trypanosoma	Reads above 500 bp	Reads above 70% coverage	Read average lenght (bp)^a^
3	388	20	9	618
5	2448	189 >>>> 60	46	620
6	317	20	15	611

^a^ Average read length for reads with >70% query coverage.

BLAST analyses of the reads were used to identify the closest taxonomic match and select a reference sequence for alignment. Because consensus reconstruction requires a single reference to anchor the reads, the species that appeared most frequently among the BLAST hits—*Trypanosoma wauwau*—was chosen for this purpose. The reads were then aligned to this reference within the 18S rRNA region of the ribosomal gene to generate a single consensus sequence.

**FIGURE 3:**
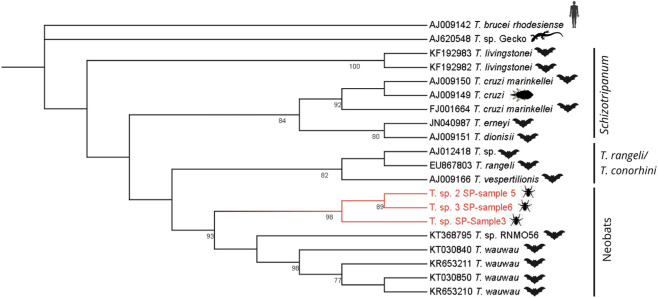
Maximum-likelihood phylogenetic tree of *Trypanosoma* spp. based on 18S SSU rRNA sequences obtained from metagenomic reads of *Strebla guajiro*. Samples 3, 5, and 6 (in red) cluster within the Neobats clade.

Using the sequence assembly approach, we recovered fragments of 534 bp (*sample3*), 823 bp (*sample5*), and 814 bp (*sample6*). In the phylogenetic analysis, the sequences from this study formed a monophyletic clade with high bootstrap support (98%), positioning evolutionary closer to *Trypanosoma wauwau* than to other species belonging to the *Schizotrypanum* group clade (Figure 3). Bootstrap values above 70 are indicated at the nodes, while sequences from this study are highlighted in red. *Trypanosoma brucei* and lizard *Trypanosoma* sequences were used as outgroups.

Pairwise similarity analyses supported the phylogenetic placement of the three sequences near *Trypanosoma wauwau*. BLASTn searches revealed that *T. wauwau* was the named species with the highest nucleotide identity (97.4%–97.8%), whereas other *Trypanosoma* species showed lower similarity values. Higher identity scores (>98–100%) were observed only for sequences classified as *Trypanosoma* sp. (unnamed), which represent genus-level matches. These quantitative results are consistent with the phylogenetic tree, where our sequences form a well-supported clade with *T. wauwau* (93% bootstrap), indicating a close but distinct relationship.

**FIGURE 4:**
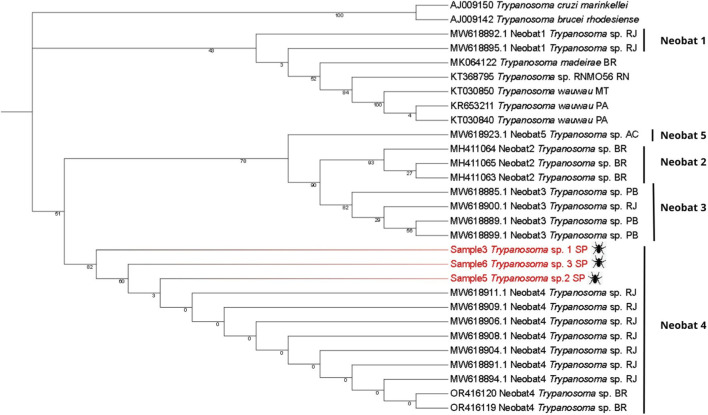
Maximum-likelihood phylogenetic tree of *Trypanosoma* spp. based on the 18S SSU rRNA region obtained from metagenomic sequences of *Strebla guajiro* (Streblidae). The tree includes five Neobat clades described for Neotropical bat trypanosomes. Samples 3, 5, and 6 (highlighted in red) cluster specifically within the Neobat 4 clade. Reference sequences are labeled with GenBank accession numbers, clade classification and geographic origin within Brazil: Rio Grande do Norte (RN), Brazil - unspecified (BR), Pará (PA), Mato Grosso (MT), Rio de Janeiro (RJ), Acre (AC), Paraíba (PB) and São Paulo (SP).

The second phylogenetic tree (Figure 4), containing only bat *Trypanosoma* spp., incorporated the findings of Alves et al. (Alves et al., 2021) regarding uncultivable *Trypanosoma* sp. sequences, which described different Molecular Operational Taxonomic Units (MOTUs), classifying them as Neobat 1, 2, 3, 4, and 5. Other Brazilian *Trypanosoma* sp. sequences available in the literature were also included to determine the alignment of the new sequences with the clades proposed by Alves et al. *Trypanosoma brucei rhodesiense* and *T.* sp. from gecko lizard were used as outgroups.

Our phylogenetic analysis confirmed the robustness of the main Neobat groupings, with significant bootstrap values of 0.9 for the Neobat2 clade and 0.8 for the relationship between Neobat2 and Neobat3, supporting the structure of 5 distinct lineages. The Neobat4 clade shows an interesting pattern in bootstrap values, with robust statistical support at basal nodes (0.823 and 0.601), but considerably low values in internal branches (0.003). Regarding geographic distribution, the Neobat4 clade is present in Rio de Janeiro and, with the three new sequences identified, in São Paulo. Neobat3 shows a broader distribution, including samples from both Paraíba and Rio de Janeiro. Available data in GenBank (NCBI) did not reveal geographic location information for sequences belonging to the Neobat2 clade, indicating a possible gap in deposited metadata.

The partial *Trypanosoma* sp. sequences obtained in this study have been deposited in GenBank under accession numbers PV945502, PV945503, and PV945504.

## Discussion

It is important to explicitly acknowledge that the results presented here are preliminary, based on a limited sample size, which limits the generalizability of the findings and prevents any ecological or statistical inferences. Therefore, all interpretations should be made with caution and require further investigation with larger and more diverse sample sets.

The detection of *Trypanosoma* in this study was possible by the untargeted nature of metagenomics, since this parasite was not the original focus and had never been found in bat fly samples before. One of the advantages of this technique is its capacity to uncover non-cultivable organisms, offering a more comprehensive view of parasite diversity in the environment.

Metagenomic detection alone does not confirm vector competence or establish the flies as biological vectors of these parasites. The presence of *Trypanosoma* DNA in Streblidae may represent transient or residual material from host blood rather than an active infection in the flies themselves, a distinction that must be acknowledged when interpreting these findings. To validate and deepen these findings, it is important to integrate other molecular tools, such as conventional PCR, and to attempt cultivation of less-studied species, followed by sequencing. This combined methodology helps not only to confirm the presence of the detected organisms, but also to generate important insights into their viability and biology, contributing to a better understanding of trypanosome ecology, especially in urban green areas.

*Trypanosoma wauwau*, first described in *Pteronotus parnellii* bats in Rondônia (Lima et al., 2015) and Pará (da Costa et al., 2016), shows phylogenetic proximity to the sequences detected in this study. These sequences were recovered from three of the five fly samples parasitizing *Carollia perspicillata*, a frugivorous bat. Previous studies in the Atlantic Forest of Rio de Janeiro (Rangel et al., 2019), Espírito Santo (Dario et al., 2017b), and the Colombian Amazon (Ramírez et al., 2014) also reported trypanosomes associated with *C. perspicillata*. In SP, previously described species in bats include *T. cruzi* (França et al., 2024) and *Trypanosoma madeirae* (Barros et al., 2019). This is the first report of sequences from a Neobat4 clade in São Paulo state. Experimentally, *T. wauwau* showed no ability to infect mammals *in vitro*, mice, or triatomines, being inactivated in the latter’s digestive system (Lima et al., 2015).

In the phylogenetic analysis of the Neobat clades, the non-monophyletic distribution of the five distinct lineages (Neobat1–5) may suggest an evolutionary history of the parasite marked by many independent events of adaptation to parasitism in bats, although this interpretation requires broader taxonomic sampling and additional molecular markers for confirmation. The bootstrap values provide variable support, since the Neobat2 clade (0.929) and its relationship with Neobat3 (0.899) stand out as well-supported taxonomic groups and Neobat3 displays a broad geographic distribution from Paraíba to Rio de Janeiro, suggesting a greater dispersal capacity or adaptability to different hosts. The Neobat4 clade exhibits a particular phylogenetic structure, with robust basal nodes (0.823 and 0.601) but low internal branching (0.003), indicating recent diversification events. Neobat1 and Neobat5 clades show distinct patterns, with Neobat1 presenting a close phylogenetic relationship with *Trypanosoma wauwau*, suggesting a possible shared evolutionary history and common ancestry.

Alves et al. (2021) identified that *Carollia* sp. trypanosomes form a unique clade, Neobat4, possibly specific to this genus, a hypothesis reinforced by the sequences from this study. The description of the Neobat clade of *Trypanosoma* sp. in Brazilian territory (São Paulo, Rio de Janeiro, Paraíba, Acre, and other unspecified locations) suggests a broader distribution of these parasites in South America, especially considering their presence in Acre state, which borders Peru and Bolivia. Further studies in other South American countries are needed to determine the true extent of this geographic distribution.

Triatomines represent proven vectors, especially *Cavernicola* spp. (De Oliveira et al., 2007), which preferentially feeds on bats in caves. In a study with *Cavernicola lenti*, protozoa with morphology similar to *T. cruzi marinkellei* was found (Barrett and Arias, 1985). In addition, in 1942, researchers documented *Cavernicola pilosa* and streblid flies collected from tree hollows that served as bat roosts, suggesting ongoing transmission (Dias et al., 1942). More recently, *Triatoma vitticeps* carrying *Trypanosoma dionisii* was reported in Espírito Santo (Dario et al., 2017b).

Beyond triatomines, experimental studies have shown that bedbugs of the species *Cimex lectularius* can act as possible vectors for some bat trypanosome species, such as *T. hedricki* and *T. myoti*. These bedbugs exhibit developmental patterns akin to those seen in *T. cruzi* within triatomines, which is especially significant given the high prevalence of these ectoparasites in urban bat roosts and their capacity to sustain transmission cycles (Paterson and Woo, 1984).

The detection of *Trypanosoma* DNA exclusively in *Strebla guajiro* specimens associated with *Carollia perspicillata* may reflect the complexity of parasite-host-ectoparasite interactions in green areas. The presence of trypanosome DNA in bat flies does not establish vector competence or biological transmission. This finding may be explained by mechanical carriagethrough occasional insect ingestion by frugivorous bats (Herrera et al., 2001), blood meal residues from direct fly ectoparasitism, or poorly understood ecological interactions. The taxonomic distinction between *Strebla guajiro* (Streblinae) and *Trichobius joblingi* (Trichobiinae) may be relevant, considering their different parasitic behaviors: *S. guajiro* primarily inhabits and moves through the host’s fur, while *T. joblingi* is typically found on membrane surfaces, though the ecological significance of this observation regarding parasite transmission remains a hypothesis requiring further investigation.

Trypanosome infections in bats are generally considered asymptomatic. However, a case of clinical disease associated with trypanosomiasis in an Australian flying fox has been described, where authors observed hemolytic anemia, jaundice, and hemoglobinuric nephrosis (Mackie et al., 2017). Pathological examination revealed numerous organisms in blood and lymphoid tissues, along with mild interstitial pneumonia and hepatic sinusoidal leukocytosis, suggesting a systemic inflammatory process. Scientific evidence shows that bats have unique mechanisms to tolerate inflammation caused by viral infections (Das et al., 2025), but the regulation of specific inflammatory responses to trypanosome infections remains poorly understood.

Data from 2011 obtained with *T. cruzi* strains isolated from urban bats showed infectivity potential for human cells, although with lower efficiency compared to established strains. Despite the low transmission risk, this finding indicates that surveillance of these parasites in bat populations remains a relevant public health concern (Maeda et al., 2011).

Climate change and land use alterations can reshape ecological interactions between bats and their ectoparasites. Such shifts may contribute to the emergence of pathogen spillover and alter the dynamics of trypanosome transmission (Eby et al., 2023).

It is important to note that the scarcity of studies on bat trypanosomes, especially in Neobats clades, limits our ability to make more comprehensive interpretations. Complementary characterization of the trypanosome diversity in bats is important to expand our understanding of these parasites’ evolution and ecology. Additional analyses using complementary molecular markers such as gGAPDH and Cytb and a broader geographic sampling are needed. Moreover, developing more sensitive molecular techniques for detecting uncultured species is essential, similar to metagenomic and metabarcoding approaches already successfully used in viral taxonomy, to better understand the evolutionary relationships within this protozoan group.

## Conclusion

This is a preliminary report representing the first report of *Trypanosoma* sp. DNA detection in flies of the Streblidae family parasitizing *Carollia perspicillata* bats, a finding made possible by shotgun metagenomics. This exploratory study also documents the first report of a Neobat 4 clade *Trypanosoma* sp. In the Atlantic Forest of São Paulo state. The identification of this potential parasite–host–ectoparasite association suggests the need for continuous continuous monitoring of these organisms, especially considering their potential impact on bat health and possible public health implications. Given the limited sample size (n = 6 flies from a single sampling event) and the exploratory nature of this metagenomic approach, broader ecological interpretations cannot be drawn at this stage. Although additional studies are needed, particularly involving expanded sampling, deeper molecular characterization, experimental validation of vector competence, and investigation of ecological interactions, this preliminary finding contributes to documenting the occurrence of this *Trypanosoma* sp. clade in streblid flies from the Atlantic Forest of São Paulo, expanding current knowledge on its geographic distribution providing initial evidence that may contribute to understanding trypanosome circulation between bats and their ectoparasites.

## Data Availability

The partial Trypanosoma sp. sequences obtained in this study have been deposited in GenBank under accession numbers PV945502, PV945503, and PV945504.
