# Supplementary material for: Metagenomics enables the first detection of Trypanosoma sp. in Streblidae (Diptera: Hippoboscoidea) parasitizing bats in São Paulo, Brazil
Source: Front Syst Biol. 2026 Jan 9;5:1721019. doi: 10.3389/fsysb.2025.1721019 (PMC12827625; doi:10.3389/fsysb.2025.1721019)
Supplement: Supplementary file 1 [file Table1.docx]

**Supplementary Table 1 - Accession numbers used in the Trypanosoma phylogenetic analyses**

1. **Tree 1 – *Trypanosoma* spp. general dataset**

| **Accession** | **Species** |
| --- | --- |
| AJ009142 | *Trypanosoma brucei rhodesiense* |
| AJ620548 | *Trypanosoma* sp. |
| KF192983 | *Trypanosoma livingstonei* |
| KF192982 | *Trypanosoma* *livingstonei* |
| AJ009150 | *Trypanosoma cruzi marinkellei* |
| AJ009149 | *Trypanosoma cruzi* |
| FJ001664 | *Trypanosoma cruzi marinkellei* |
| JN040987 | *Trypanosoma erneyi* |
| AJ009151 | *Trypanosoma dionisii* |
| AJ012418 | *Trypanosoma* sp. |
| EU867803 | *Trypanosoma rangeli* |
| AJ009166 | *Trypanosoma vespertilionis* |
| KT368795 | *Trypanosoma* sp. RNMO56 |
| KT030840 | *Trypanosoma wauwau* |
| KR653211 | *Trypanosoma wauwau* |
| KT030850 | *Trypanosoma wauwau* |
| KR653210 | *Trypanosoma wauwau* |

1. **Tree 2 – *Trypanosoma* sp. dataset**

| **Accession** | **Species** |
| --- | --- |
| MK064122 | *Trypanosoma madeirae* |
| KT368795 | *Trypanosoma* sp. RNMO56 |
| KT030840 | *Trypanosoma wauwau* |
| KR653211 | *Trypanosoma wauwau* |
| KT030850 | *Trypanosoma wauwau* |
| AJ009150 | *Trypanosoma cruzi marinkellei* |
| AJ009142 | *Trypanosoma brucei rhodesiense* |
| MW618892.1 | *Trypanosoma* sp. |
| MW618895.1 | *Trypanosoma* sp. |
| MW618923.1 | *Trypanosoma* sp. |
| MH411065 | *Trypanosoma* sp. |
| MH411064 | *Trypanosoma* sp. |
| MH411063 | *Trypanosoma* sp. |
| MW618900.1 | *Trypanosoma* sp. |
| MW618889.1 | *Trypanosoma* sp. |
| MW618885.1 | *Trypanosoma* sp. |
| OR416120 | *Trypanosoma* sp. |
| MW618908.1 | *Trypanosoma* sp. |
| MW618909.1 | *Trypanosoma* sp. |
| MW618906.1 | *Trypanosoma* sp. |
| MW618904.1 | *Trypanosoma* sp. |
| MW618911.1 | *Trypanosoma* sp. |
| OR416119 | *Trypanosoma* sp. |
| MW618894.1 | *Trypanosoma* sp. |
| MW618891.1 | *Trypanosoma* sp. |
